# Extracting Insights From Temporal Data by Integrating Dynamic Modeling and Machine Learning

**DOI:** 10.3389/fphys.2020.01012

**Published:** 2020-08-12

**Authors:** Richard Ballweg, Kristen A. Engevik, Marshall H. Montrose, Eitaro Aihara, Tongli Zhang

**Affiliations:** Department of Pharmacology and Systems Physiology, University of Cincinnati College of Medicine, Cincinnati, OH, United States

**Keywords:** gastric epithelium, organoids, actin, restitution, computational model

## Abstract

Biological processes are dynamic. As a result, temporal analyses are necessary to fully understand the complex interactions that occurs within these systems. One example of a multifaceted biological process is restitution: the initial step in complex wound repair. Restitution is a dynamic process that depends on an elegant orchestration between damaged cells and their intact neighbors. Such orchestration enables the quick repair of the damaged area, which is essential to preserve epithelial integrity and prevent further injury. High quality dynamic data of the cellular and molecular events that make up the gastric restitution process has been documented. However, comprehensive dynamic models that connect all relevant molecular interactions to cellular behaviors are challenging to construct and experimentally validate. In order to efficiently provide feedback to ongoing experimental work, we have integrated dynamical modeling and machine learning to efficiently extract data-driven insights without incorporating detailed mechanisms. Dynamical models convert time course data into a set of static features, which are then subjected to machine learning analysis. The integrated analysis provides data-driven insights into how repair might be regulated in individual gastric organoids. We have provided a “*proof of concept*” of how such an analysis pipeline can be used to analyze any temporal dataset and provide timely data-driven insights.

## Introduction

Temporal change is an essential feature of biological systems. An example of this is phenomena is found within the gastric epithelium. The epithelium closely regulates tissue integrity and rapidly responds to insult. When small injury or apoptosis occurs within the epithelial layer, dead or dying cells depart from the epithelial layer and neighboring cells immediately repair the damaged site by stimulating cell migration to close the gap (Xue et al., 2010; Xue et al., 2011; Aihara et al., 2013). This rapid repair response, known as restitution, does not involve proliferation and is sufficient to restore the epithelial barrier function (Ito et al., 1984; Lacy, 1995; Aihara et al., 2013; Aihara and Montrose, 2014). Restitution is a biologically important process to maintain epithelial integrity and prevent further expanding injuries. Numerous studies have identified effectors necessary for proper epithelial repair, including actin dynamics (Aihara et al., 2018). While fundamental repair mechanisms have been identified, the details behind these epithelial signaling cascades and sequences remain largely unclear.

Evaluating the mechanism behind restitution *in vivo* has proved difficult due to systemic limitations and limited tools to manipulate and monitor repair. While prior *in vitro* studies have identified potential pathways mediating repair, these findings largely rely on cancer derived cell line models which differ from the native tissue (Saeidnia et al., 2015). The introduction of the novel *in vitro* organoid model has allowed for growth of non-cancerous epithelial cells that contain cell types of the native tissues (Mahe et al., 2013; Schumacher et al., 2015a; Engevik et al., 2018). The organoid model has provided a three dimensional primary cell culture system which resembles the normal gastric epithelium, exhibiting similar gene expression patterns and functions as the native tissue (Schumacher et al., 2015a; Engevik et al., 2016; Aihara et al., 2018; Engevik et al., 2019). As a reductionist gastric restitution model, organoids can provide better insight to the innate epithelial cell response during damage in real time (Aihara et al., 2018; Engevik et al., 2019; Hanyu et al., 2019). *In vitro* organoid experimental methods produce high quality data sets that require extensive analysis to reveal underlying cellular events. As it is difficult to statistically compare two sets of dynamical data, current analysis is typically performed by comparing treatment groups at specific time points within the data, or specific features of the data such as a peak value that can be easily determined in each sample (Xue et al., 2010; Xue et al., 2011; Paek et al., 2016; Aihara et al., 2018; Engevik et al., 2019). The heterogeneity of organoids, which harbor multiple cell types, also adds to the complexity of analysis. Furthermore, detailed knowledge of the molecular control network is often unavailable for many biological systems, which generates a challenge if novel systems or pathways are studied. To address the above challenges, we have developed a novel analytical pipeline that converts single cell temporal microscopy data sets into data-driven, dynamic models that are then followed up with machine learning analysis. Here, we demonstrate the capability of this pipeline by using gastric epithelial repair as one example.

We have previously established gastric organoids, grown from epithelial stem cells of native mouse tissue, to investigate the innate epithelial restitution response (Schumacher et al., 2015a; Aihara et al., 2018; Engevik et al., 2018; Engevik et al., 2019). Using time course two-photon confocal microscopy of gastric organoids generated from human GFP-actin (HuGE) transgenic mice, we demonstrated that actin assembly occurs within the migrating cells neighboring the injured site, followed by recruitment of myosin II to provide cell contractility, which is regulated by RhoA/Rho associate kinase (ROCK) (Aihara et al., 2018). Actomyosin dynamics are particularly important in providing the force necessary to exfoliate the damaged cell away from the epithelial layer and to allow the neighboring cells to cover the denuded area (Stricker et al., 2010; Levayer and Lecuit, 2012; Aihara et al., 2018). While several actomyosin dynamic components and effectors have been identified as necessary for the role of actin in repair, the overall molecular mechanism remains unknown. Several studies have examined the molecular mechanisms of epithelial repair using computational models (Sherratt and Murray, 1990; Dale et al., 1994; Wearing and Sherratt, 2000; Tremel et al., 2009). Previously, we applied experimental data to a computational model and elucidated the role of force generated by actin dynamics during repair (Aihara et al., 2018).

**FIGURE 1:**
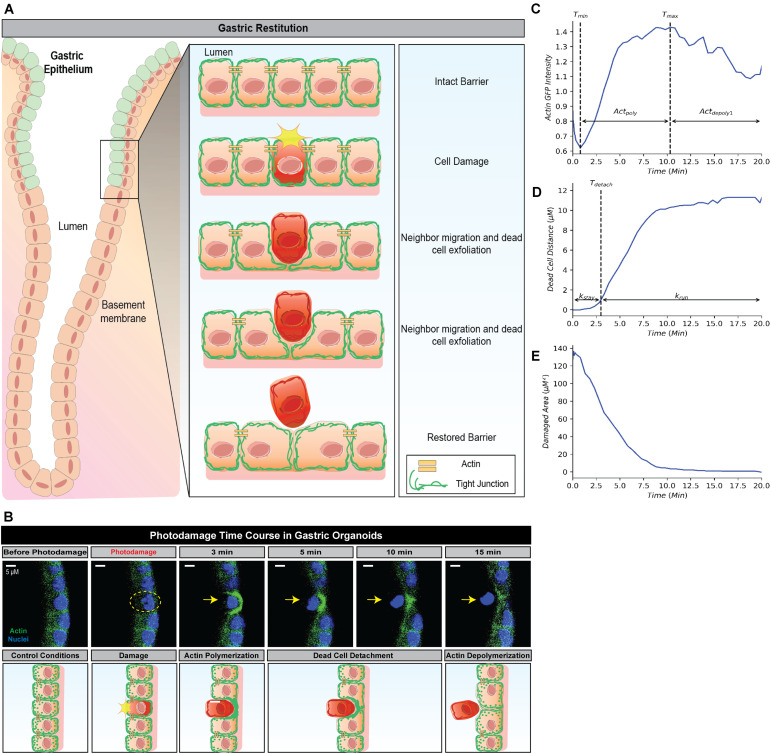
Simplified framework to recapture gastric repair dynamics. **(A)** Illustration of gastric epithelial repair response to single cell damage *in vivo*. Following cell death or damage, intact cells neighboring the damaged cell undergo actin polymerization which promotes migration toward the area of damage in order to fill the denuded area and assist in exfoliating the damaged cell away from the epithelium. Through this actin polymerization and subsequent migration, the neighboring cells ensure there are no denuded areas and restore continuity to the epithelial barrier. *In vivo*, these steps are important to promote epithelial homeostasis and prevent further damage to the epithelium and underlying mucosal layers. **(B)** Representative image (above) and illustration (below) of epithelial repair response to single cell damage *in vitro* in gastric organoids. Following single cell damage by high power laser (known as photodamage), the damaged cell (above; indicated by yellow dashed circle and arrow) loses GFP-fluorescence while intact neighboring cells show increased GFP-actin in green (indicating actin polymerization) in the lateral region closest to the damage site. The neighboring cells migrate and fill in the damage area, followed by the exfoliation of the damaged cell toward the lumen (dead cell detachment). This results in actin depolymerization and ultimately a restored epithelial barrier function. **(C)** Temporal trajectory of actin following damage (*t* = 0 min), actin first begins to degrade to a minimum where it then accumulates to a maximum level (actin polymerization) before degrading to a steady state (actin depolymerization). *T*_*min*_ and *T*_*max*_ indicate the time at which actin reaches the minimum and maximum values, respectively. **(D)** Temporal trajectory of the dead cell following damage (*t* = 0). The cell begins to move away at a constant rate before detaching from its neighboring cells; following detachment, the dead cell rapidly moves to a maximum distance from the damage site. *T*_*detach*_ indicates the time of detachment. **(E)** Temporal trajectory of the damaged area following damage (*t* = 0). Following single cell damage, the maximum damage size is reached at *t* = 0 and decreases in size overtime indicating repair.

To further connect molecular level events with outward cellular behaviors of this system, individual models were constructed to describe each step of the repair process (actin polymerization, dead cell movement and restitution) from experimental values detailed in Aihara et al. (2018). Aihara et al. (2018) applied a high power laser to gastric organoids, resulting in the damage and death of a single cell within an organoid (known as photodamage) (Section “Methods and Materials”). During subsequent high resolution imaging of the organoid, the role of actin was assessed over time by measuring actin polymerization and depolymerization (based upon actin GFP intensity) in neighboring cells. Additionally, measurements were made of the damaged area (the size of the damaged area based upon absence of GFP fluorescence in the damaged/dead cell), and dead cell distance (based upon the movement of the dead cell away from the damage site) (Figure 1B; Aihara et al., 2018). Using these experiment-derived parameters (Aihara et al., 2018), which summarize the dynamical characteristics of individual gastric organoids, we assessed how different treatments affect system dynamics. Random forest analysis of these data-derived parameters made clear the dominant features that link the molecular level events to the cellular level behaviors. Our analysis provides deep insights on how the molecular and cellular events control damage repair, which will move forward the development of targeted therapeutics for the native tissue repair.

Since our pipeline can be applied to analyze individual time course data sets to extract insights across various systems to enhance our biological understanding, we expect that our analysis pipeline can be a useful tool to provide efficient analysis and promote effective interdisciplinary collaboration.

## Results

### Extracting Cell-Specific Features From Dynamical Data

Restitution is an intrinsic function in gastric epithelial cells and generally involves the following events: (1) orderly reorganization of cell-cell and cell-substratum contact to allow viable cells to migrate from their site of origin, (2) cytoskeletal reorganization, including the formation of lamellipodia to promote cell movement, and (3) signal production to promote dead cell exfoliation, viable cell migration and prevent cell death within the viable cells (Aihara et al., 2017; Aihara et al., 2018). All steps are essential to restore epithelial barrier function (Figures 1A,B). Time course microscopy of high power laser induced single cell damage and subsequent repair suggests that the timing of these events is integral to the overall temporal trajectory for each measurement: actin GFP-intensity, dead cell distance, and damaged area (Figures 1C–E). In GFP-actin gastric organoid experiments, the time at which actin reaches minimal fluorescence (***T***_*min*_), indicates the elimination of actin within the damaged cell and the initial time where F-actin begins to accumulate within the neighboring cells (Figure 1C). This initial decay is controlled by the parameter (***Act***_*depoly*2_). The time at which actin reaches peak fluorescence (***T***_*max*_) determines when actin begins to contract, assuming that F-actin dissociates into G-actin at this point while the sustained increase of fluorescence likely occurs due to cellular contraction (Figure 1C). The increase of actin to its maximum is controlled by the parameter ***Act***_*poly*_. The decrease of actin to its steady state (***Act***_*SS*_) is controlled by the parameter ***Act***_*depoly*2_. During the repair process, dead cell exfoliation occurs following detachment from the neighboring cells (***T***_*detach*_, Figure 1D), where upon the damaged cell moves rapidly to a maximal distance (dead cell distance, ***DCD***_*max*_) from the monolayer that contains all the viable cells. The rate controlling this movement is defined as ***k***_*run*_. Additionally, the damaged area is repaired in a pattern that follows an exponential decay curve controlled by the parameter ***T***_*repair*_ (Figure 1E).

Due to the heterogeneous nature of the cells within gastric organoids, which reflect the gastric epithelium, each experiment results in a different time dependent trajectory for the three characteristics described above. To properly align these cellular and molecular events into a time dependent chain of events, we chose to use a hybrid model (section “Methods and Materials”). The hybrid model best describes the dynamics of each event as the repair process involves continuous time dependent changes and discrete timing events (Bartocci and Lio, 2016). Hence, these dynamical models decompose the time dependent microscopy data for each individual experiment into a collection of cell-specific features which describe the dynamics observed as a single train of events for the cell under study.

### Assembling Physiological Repair Data Within a Single Dynamical Framework

**FIGURE 2:**
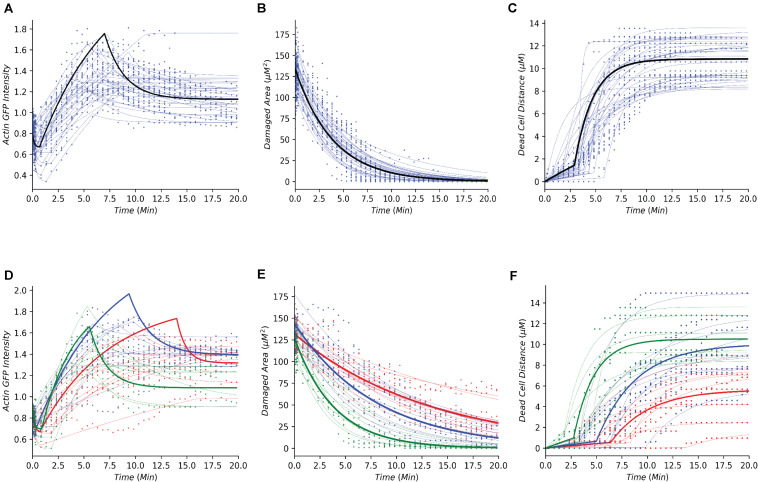
Simple dynamical models recapture the gastric repair process in control and perturbed scenarios. **(A–C)** Temporal profiles of actin, damaged area, and dead cell movement, respectively. Blue dots represent experimental data from control (untreated) organoids that have been previously reported in Aihara et al. (2018). Blue dashed lines represent model simulations for individual organoid outcomes, and black lines represent the simulation of an average control cell. **(D–F)** Temporal profiles of actin, damaged area, and dead cell movement in control (green), or treatment with Blebbistatin at high dosage (red) or low dosage (blue). Dots represent data collected from the individual experiments in Aihara et al. (2018). Dashed lines represent model simulations and solid lines show the simulation of an average cell.

Following the development of the data-driven models, we tested whether this modeling framework could recapture the temporal dynamics of gastric repair within control experiments. Both the model and data indicated that actin dynamics undergo a three-stage response to cellular damage: actin polymerization and depolymerization (actin-GFP intensity), decrease of damage area (damaged area), and exfoliation of the dead cell from the epithelial monolayer (dead cell distance). In the initial stage, actin within the damaged cell diminishes (indicated by loss of GFP fluorescence, Figure 1B) at an unknown rate before it reaches a minimal level. This rate is unknown as this rapid event cannot be captured following damage due to the imaging time course. Once the minimal level is reached, actin polymerization increases in the neighboring cells followed by these neighboring cells migrating to cover the damage area. During this set of events, actin polymerization arrives at a maximal (peak) level. Following its peak, actin decreases (actin depolymerization) until it reaches steady state. Using our model, we simulated the actin trajectory at a single cell level for more than 20 experiments (blue dashed lines Figure 2A and Table 1). The simulated dynamics agreed reasonably well with the measured actin levels (blue dots Figure 2A) and recaptured all three stages of actin’s response to damage (actin polymerization, peak, and actin depolymerization). Simulating the average actin dynamics (black line Figure 2A) showed that the average model simulation fell within the region bounded by the experimentally observed actin levels, indicating that the overall model fit was reasonable.

**TABLE 1:** Average parameter values and respective units from control organoid experiments shown in Figures 2A–C.

Parameter	Value (Mean ± SD)	Units
ACt_*depolyl*_	6.024 ± 3.557	min^–1^
Act_*depoly2*_	0.616 ± 0.39	min^–1^
ACt_*poly*_	0.191 ± 0.171	min^–1^
ACt_*max*_	2.484 ± 0.944	RLU
ACt_*min*_	0.696 ± 0.115	RLU
Act_*ss*_	1.083 ± 0.168	RLU
DCD_*max*_	10.507 ± 2.03	μm
K_*repair*_	0.255 ± 0.08	μm^2^ min^–1^
K_*run*_	0.462 ± 0.287	μm min^–1^
K_*stay*_	0.035 ± 0.023	μm min^–1^
T_*detach*_	2.749 ± 1.134	Min
T_*max*_	5.534 ± 1.185	Min
T_*min*_	0.886 ± 0.726	Min

Experimental data indicated that repair of the damaged area, as indicated by the decrease in damage size over time, is a single-stage process that appears to follow an exponential decay curve (blue dots, Figure 2B and Table 1). With our model, we simulated the repair of the damaged area at a single cell level for more than 20 experiments (blue dashed lines Figure 2B). The simulated repair of the damaged area agreed reasonably well with the experimentally observed repair (blue dots Figure 2B). Furthermore, simulation of the average repair of the damaged area (black curve Figure 2B) we observed that the overall model fit was able to recapture the experimental observations.

In addition to actin dynamics and repair of the damaged area, the experimental data also indicated that dead cell exfoliation (dead cell distance) is a two-stage process whereupon the dead cell is slowly moved away from the monolayer at a constant rate and then at a specific time (**T**_*detach*_) the cell is fully detached and the dead cell accelerates until it reaches steady state distance from the damaged area (blue dots, Figure 2C and Table 1). Using our model, we simulated the distance traveled in each experiment (blue dashed lines, Figure 2C). The model was able to recapture the initial constant velocity, acceleration and steady state of the experimental observation. The average simulated dead cell was well within the region bounded by the experimental results indicating that the derived parameters were reasonable (black line, Figure 2C).

The ability of our modeling framework to recapture these key features of normal gastric repair suggests that the model assumptions and the derived parameter values are reasonable. With the affirmation that our model assumptions align with the cellular events under normal conditions, we then examined whether the same approach could be used to study perturbation experiments.

### Organization of the Perturbed Dynamics Within the Dynamical Framework

For perturbation studies of the model, we chose a common myosin inhibitor (Blebbistatin) which shows a clear dose dependent response on the temporal dynamics of gastric repair (Figures 2D–F; Aihara et al., 2018). Using the same data-driven approach (Section “Methods and Materials”), we fit single cell models for the control experimental group (green dots Figures 2D–F), along with high dosage and low dosage Blebbistatin experimental groups (red and blue dots, respectively, Figures 2D–F). Our models faithfully recaptured the actin dynamics, repair of damage, and dead cell distance of all three treatment groups of control, high dosage Blebbistatin and low dosage Blebbistatin (dashed lines, Figures 2D–F). As in the control case, average cells (thick lines, Figures 2D–F) were well within the regions bound by their respective data sets, indicating that the derived parameters were within reason. The model was also able to recapture the dose dependent response of the Blebbistatin treatments. For instance, a low dose of Blebbistatin causes a decrease in repair compared to control experiments, which is further decreased by a higher dose of Blebbistatin. This phenomenon was also observed in the maximal distance reached by the dead cell. In concordance with Blebbistatin, the computational framework was able to recapture the remaining experimental data published by Aihara et al. (2018). As the model was able to recapture such a wide array of experimental scenarios, this method could be considered a solid framework to study the gastric repair process *in silico* and additional analysis of the derived parameters can offer mechanistic insights into the key events that characterize the restitution process.

### Chord Plotting Reveals the Dominant Effect of the Perturbations

**FIGURE 3:**
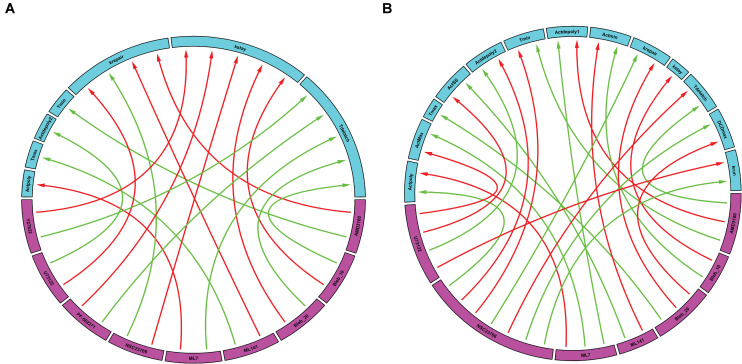
Data-driven analysis provides insights into the control of gastric repair. Arrows originate from the drugs (pink boxes) and point to a feature of the model (blue boxes). **(A)** Chord plot of the maximal interactions of each drug treatment to model parameters. Each drug can inhibit or activate a model feature. A green arrow represents the parameter that is most activated by a drug. A red arrow indicates the feature that is most inhibited by a drug. **(B)** Chord plot of the maximal enhancement or inhibition of each model feature by any drug treatment. Each model feature (pink boxes) can be inhibited or activated by a drug treatment (blue boxes). A red arrow indicates that the treatment has the highest inhibition of the model feature when compared to all other treatments. A green arrow indicates that the treatment has the highest activation of a model feature when compared to all other treatments. It is important to note that there are differences in the parameters and treatments displayed when comparing figures, **(A,B)**. In **(A),** not all parameters are shown as some might not be greatly affected by the treatments being examined; whereas in **(B),** not all treatments are shown as they might not have large effects on the model parameters when compared to the rest of the treatments.

Following simulation for each treatment group, we aggregated both the model derived and data-driven parameters to look for any additional insight into the potential effect of various inhibitors previously tested (Aihara et al., 2018) on the events that control the gastric repair process. For each treatment group, the percent change between the model parameters of the treatment and control experiments was calculated. The parameters exhibiting the highest positive change and the parameters values with the highest negative change were then plotted on a chord plot (Figure 3), which connects each treatment with the mechanistic properties they most strongly regulate (increases indicated by green arrows; decreases indicated by red arrows). By plotting interactions in this form, we provide a concise picture into the effect of each inhibitor during restitution. For instance, NSC23766 (a selective Rac1 inhibitor) causes a large increase in *k_repair_* (green arrow, Figure 3A), indicating that the treatment causes an increase in the rate of damage repair when compared to controls. In contrast, ML-141 (a potent, selective inhibitor of Rho family GTPase cdc42) decreases the rate of gastric repair, while also increasing *T_max_*, (green arrow, Figure 3A), indicating that the rate of gastric repair may be associated with an increase in *T_max_* compared to controls. ML-141 has been shown to decrease actin polymerization in airway epithelial cells (Ferru-Clement et al., 2015). Furthermore, we observe that both dosages of Blebbistatin affect the same mechanistic control parameters (*k_stay_* and *T_detach_*), consistent with the dose dependent response of this treatment shown in Figures 2D–F.

We also plotted the overall maximum enhancement or inhibition of each model parameter (Figure 3B) compared to treatment. Each parameter contains two inputs: the maximum enhancement effect and the maximum inhibitory effect. Note that ***T***_*max*_ and ***k***_*stay*_ only have a single input as all treatment groups were shown to increase ***T***_*max*_ or decrease (***k***_*stay*_) control parameters. From this plot, we can deduce which drugs have the greatest overall effect (compared to all other drugs) on any of the model’s features. For instance, AMD3100 (a CXCR4 inhibitor) showed the largest overall enhancement effect on ***T***_*detach*_ (the parameter controlling the time at which the dead cell loses connection with its neighbors). Additionally, AMD3100 had the largest inhibitory effect compared to all other drugs on ***Act***_*depoly*1_ (parameter for actin depolymerization). Based on this data and our observations of the effects of drugs on individual parameters, we are able to observe which treatments have the most profound effects on the overall repair mechanism. Interestingly, NSC23766 caused the most changes to the model features, with a total of 7 connections, indicating that NSC23766 might have the most profound effect on the overall system compared to all other treatments in the plot. On the basis of such results, we can then identify the role of Rac1 (inhibited by NSC23766) as a promising target during repair for future studies.

### Random Forest Reveals the Control of Damage Repair and Dead Cell Exfoliation by Actin

**FIGURE 4:**
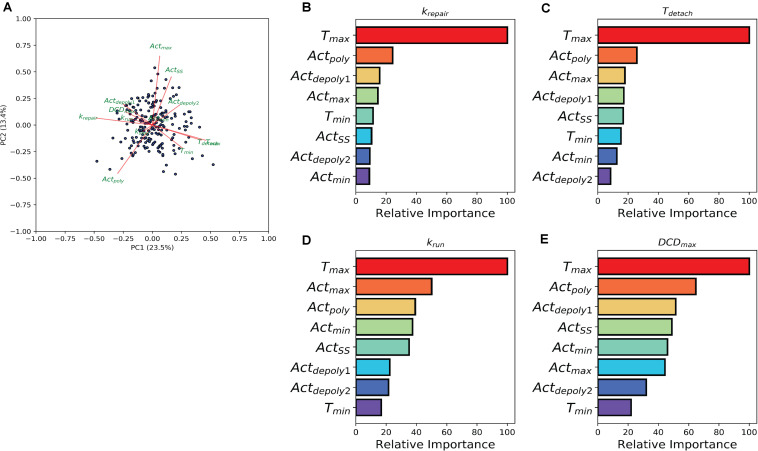
Machine learning links timing events between molecular and cellular level events. **(A)** PCA analysis biplot of the model parameters. Dots represent samples plotted based on the first two principal components. Red arrows represent the loadings from the PCA. **(B–E)** Bar plots depicting Random Tree variable importance analysis for the indicated parameters: **(B)** repair rate (*k*_*repair*_), **(C)** detachment time (*T*_*detach*_), **(D)** rate at which the dead cell is pushed out by its neighbors (*k*_*run*_), and **(E)** maximum dead cell distance (*DCD*_*max*_). Axis is scaled 0 – 100, where 100 represents parameters that are highly significant in the random forest model and 0 represents insignificant parameters.

The previous analysis demonstrated the effect of individual treatment on actin accumulation, repair of the damaged area, and dead cell exfoliation, but does not address the potential connections between these processes. To investigate how the events can be connected, we then analyzed whether the molecular level events (actin polymerization and depolymerization) might be associated with the cellular level behaviors (damage repair, cell exfoliation). While prior analysis separated experiments into groups based upon treatment scenarios, we carried out a pooled study whereupon all experiments (treated and controls) are combined into a single population which exhibited heterogeneous actin dynamics. To assess the correlation amongst the model parameters we performed a principal component analysis (PCA). A biplot of the analysis (Figure 4A) points to a multitude of correlations between the parameters controlling actin dynamics and parameters controlling cellular behaviors. In particular, there is a strong positive correlation between **T**_*max*_ and **T**_*detach*_. Additionally, there were strong negative correlations between **Tmax** and parameters controlling dead cell exfoliation (**k**_*run*_, **DCD**_*max*_) and the parameter controlling the repair of the damaged site (**k**_*repair*_).

The relationship between the parameters which describe actin dynamics and the rate at which the damaged site is repaired were further assessed using a tree-based regression method (Random Forest regression). An ensemble of regression trees was built with the data-driven parameters and model derived rates for actin, which we then used to predict the corresponding restitution rates from both a training and validation cohort (section “Methods and Materials”). The random forest performed well in predicting restitution rates for both training and validation cohorts (*R*^2^ Training = 0.93 and *R*^2^ Validation = 0.34) indicating a robust statistical model which could be used for future study. Due to the strength of the Random Forest Regression, we performed a variable importance analysis to identify which actin parameters made significant contributions to the statistical model. This analysis revealed that ***T***_*max*_ (the time at which actin reaches its peak) plays a significant role in determining the rate of repair (Figure 4B), suggesting a potential mechanistic link between the two processes. The random forest analysis was then repeated for each of the parameters describing dead cell extrusion (***k***_*stay*_, ***T***_*detach*_, ***k***_*run*_, and ***DCD***_*max*_). Interestingly, ***T***_*max*_ was revealed to be important for ***T***_*detach*_, ***k***_*run*_ and ***DCD***_*max*_ (Figures 4C–E); indicating that the time of peak actin accumulation is a strong predictor for the dynamics of the cell exfoliation process. Hence without explicitly incorporating any knowledge, our data-driven analysis reveals a critical role for actin polymerization timing in determining the cellular level behaviors involved in the gastric repair process.

## Discussion

In this work, we have developed a computational pipeline that can efficiently examine key factors involved in *in vitro* restitution using temporal data collected from time lapse confocal microscopy of gastric organoids (Aihara et al., 2018). Our approach reveals that the timing of actin cytoskeleton rearrangement greatly influences the repair of the damaged area, as well as the exfoliation of the damaged cell. We predict that these functions are critical for the healing process and have implications in situations, such as *Helicobacter pylori* infection, where wound healing is delayed (Aihara et al., 2014; Hanyu et al., 2019). *In vitro* organoid studies in epithelial repair (Aihara et al., 2018; Engevik et al., 2019; Engevik et al., 2020) have been demonstrated to be similar to native tissue as they demonstrate (1) importance of actin dynamics in cells neighboring damage sites (Aihara et al., 2018), (2) increased calcium mobilization, which is dependent upon trefoil factor 2 signaling during repair (Xue et al., 2010), and (3) role of sodium hydrogen exchanger 2 downstream of the trefoil factor 2 pathway in repair (Xue et al., 2011). These *in vitro* studies identified features intrinsic to the epithelium and is reflective in native tissue. Utilization of data sets from organoid experiments, which exhibit similar responses to infection and damage as native tissue (Schumacher et al., 2015a, b; Aihara et al., 2018), provides a reductionist platform to study repair at the cellular level with the potential to better translate to *in vivo* events.

Current methods of analysis for epithelial repair, such as exponential analysis of the damaged site or comparison between specific time points, offer statistical differences between experimental groups (Aihara et al., 2018) and indicate important aspects of repair. However, these techniques have not taken full advantage of all the temporal data or revealed the underlying heterogeneity between individual cells. On the other hand, though mechanistic modeling is suitable for dynamical data sets and numerous mathematical models of varying complexity have indeed provided valuable insights on epithelial repair and/or wound repair (Dale et al., 1994; Wearing and Sherratt, 2000), the development of these models often requires significant amount of time and resource.

To facilitate theory-experiment collaboration with timely feedbacks, we have proposed to integrate simple dynamical models with following up machine learning analysis. This approach allowed us to utilize the entire data set while incorporating minimal assumptions (time dependent behaviors for each process and that all processes are independent) about each process. By using this data-driven approach, the model parameters are derived directly for each individual cell. In doing so, each cell can be encoded as a group of dynamic features that have been extracted directly from data and are associated with each other during analysis. This method decomposes the time course data into static features which are suitable for downstream analysis. Machine learning algorithms such as random forest regression can then be used to compare differences between individual cells and reveal what features are vital for the gastric restitution process. This combination provides the foundation to look further into the cellular and molecular events essential for proper repair.

Biological systems are very complex and proper understanding of them is beyond the capability of any single method, and we believe the new area of systems biology calls for the synergistic integration of all available tools. In our example we have shown that a proper combination of real time microscope, cutting edge organoid culture, dynamical modeling, and machine learning can produce efficient insights on a new biological system. We believe that such combination can be applicable to any biological systems and will facilitate deep understanding of them.

## Materials and Methods

### Experimental Data Acquisition

The experimental data used for this study has previously been published (Aihara et al., 2018). Gastric organoids were generated from HuGE (Human GFP-Actin Expressing) transgenic mice as previously described (Schumacher et al., 2015a; Aihara et al., 2018). Using a two-photon confocal microscope (Zeiss LSM 510 NLO), images of gastric organoid nuclei (Hoechst 33342; Ti-Sa excitation 730 nm, emission 435–485 nm) and actin (GFP; excitation 488 nm, emission 500–550 nm) were collected (Aihara et al., 2018). After collecting a set of control images, a small region (∼5 μm^2^) of a cell within the gastric organoid was repetitively scanned for 2–3 s at high laser power resulting in single cell damage (photodamage) (Aihara et al., 2018). For inhibitory experiments, gastric organoids were incubated with drugs at least 1 h prior to experiments: AMD3100 (1 μM, Sigma), U73122 (10 μM, Enzo life sciences), ML7 (10 μM, Calbiochem), Blebbistatin (20 μM or 10 μM, Sigma), NSC23766 (50 μM, Cayman), PF-562271 (1 μM, gift from Dr. James E. Casanova, Univ Virginia), ML141 (20 μM, Calbiochem), and Y27632 (20 μM, Enzo Life Sciences). The parameters measured over time from the collected gastric organoid images include: actin polymerization based upon GFP intensity (Aihara et al., 2018; Hanyu et al., 2019), size of damage area (Aihara et al., 2018; Engevik et al., 2019; Hanyu et al., 2019; Engevik et al., 2020), and dead cell distance based upon movement of damage cell nuclei away from the site of damage (Aihara et al., 2018; Engevik et al., 2019; Hanyu et al., 2019). The damage-repair cycle was measured once per gastric organoid, and outcomes of at least four different gastric organoids were compiled for each experiment.

### Model Development

To recapture each of the experimentally observed features we use a collection of piecewise linear ordinary differential equations (ODE). Each feature is modeled as an independent process and is controlled by a single ODE.

### Actin Model

$$\frac{dAct}{dt}=Act_{poly}*\left(Act_{max}-Act\right)-Act_{depoly2}*$$

$$\left(Act-Act_{SS}\right)-Act_{depoly1}*\left(Act-Act_{min}\right)$$

Where *Act*_*poly*_ represents the rate of actin polymerization, which is only on between *Act*_*max*_the maximum value of actin, *Act_*depoly*2_* the rate at which actin degrades following the actin peak, *Act*_*SS*_ the steady state that actin degrades to. *Act*_*min*_ is the minimal actin level for each cell and *Act*_*depoly*1_ is the rate that actin degrades to *Act*_*min*_. Rate constants are turned on or off dependent on certain timing events with: *Act*_*depoly*1_ on between *t* = 0 and *t* = *T*_*min*_; *Act*_*poly*_ on between *t* = *T*_*min*_ and *t* = *T*_*max*_ and *Act*_*depoly*1_ on between *t* = *T*_*max*_ and the end of the simulation.

### Damaged Area Model

$$\frac{dDA}{dt}=-k_{repair}*DA$$

Where *k*_*repair*_ represents the rate of repair of the damaged area.

### Dead Cell Distance

$$\frac{dDCD}{dt}=k_{stay}+k_{run}*\left(DCD_{max}-DCD\right)$$

Where *k*_*stay*_ represents the background force acting on the dead cell, *k*_*run*_ is the rate at which the dead cell is pushed out by neighboring cells and *DCD*_*max*_ is the maximum distance traveled by the dead cell. *k*_*run*_ is turned on when the dead cell loses connection with its neighboring cells (*T*_*detach*_).

### Model Parameterization

Parameters controlling the timing events (*T*_*max*_, *T*_*min*_, and *T*_*detach*_) along with *Act*_*min*_ and *Act*_*SS*_ were extracted directly from the data for individual organoids in all treatment groups. To extract rate constants and *Act*_*max*_ (assumed to be theoretical), the ODEs were first separated into different events and integrated with respect to time. An evolutionary algorithm was then used to minimize the sum of squares between model simulation for each experiment from Aihara et al. (2018). This process was repeated for each experiment, generating a cohort of parameter sets, each representing an individual organoid.

### Principal Component Analysis

To understand the potential relation among individual experiments and correlation among derived model parameters in an unsupervised approach, we performed a Principal Component Analysis (PCA). All of the derived parameters were considered and scaled prior to analysis. The PCA was performed using the PCA function within the scikit-learn package.

### Random Forest Analysis

To understand how the parameters that control actin influence the cellular behaviors (Dead Cell Movement, Repair) we used Random Forest regression, an ensemble tree based regression method. This method was chosen as it does not require knowledge of how the predictors should be combined. The actin parameters for all groups (treatment and controls) were used as predictors while the parameters controlling cell behaviors (*k*_*repair*_, *T*_*detach*_, *k*_*stay*_, *k*_*run*_, and *DCD*_*max*_) were used as response variables. The data was then randomly split into Training and Validation sets and the Random Forest model was built using the default settings contained within the scikit-learn package. A variable importance analysis was performed to assess the importance of each predictor (parameters controlling actin dynamics) on the response variables (parameters controlling damage repair or cell exfoliation). This was done using the default calculation given within the scikit-learn package, which assesses the Gini importance of each predictor. The importance values were then scaled to the maximal importance and compared.

### Packages Used

All ordinary differential equations were implemented in Python and solved using ODEint from the scipy package^1^. Chord plots were generated using Circlize (Gu et al., 2014) implemented in *R*^2^. Random forest regression was done using the standard package implemented in scikit-learn^3^.

## Data Availability Statement

The raw data supporting the conclusions of this article will be made available from the authors upon request.

## Author Contributions

RB, KE, MM, EA, and TZ conceived and designed the research. RB, KE, EA, and TZ performed the experiments and analyzed the data. All authors edited and revised the manuscript. MM, EA, and TZ obtained research funding for experiments and computational modeling.

## Conflict of Interest

The authors declare that the research was conducted in the absence of any commercial or financial relationships that could be construed as a potential conflict of interest.

## Footnotes

1 https://www.scipy.org/

2 https://www.r-project.org/

3 https://scikit-learn.org/stable/
